# Oxygen uptake efficiency slope in healthy normal weight young males: an applicable framework for calculation and interpretation

**DOI:** 10.7717/peerj.13709

**Published:** 2022-07-13

**Authors:** Lavinia Falcioni, Laura Guidetti, Carlo Baldari, Maria Chiara Gallotta, Marco Meucci

**Affiliations:** 1 Department of Movement, Human and Health Sciences, University of Rome “Foro Italico”, Roma, Lazio, Italy; 2 Department of Unicusano, Niccolò Cusano University, Roma, Lazio, Italy; 3 Department of Theoretical and Applied Sciences, eCampus University, Novedrate, Lombardia, Italy; 4 Department of Physiology and Pharmacology “Vittorio Erspamer”, Sapienza University of Rome, Roma, Lazio, Italy; 5 Department of Health and Exercise Science, Appalachian State University, Boone, North Carolina, United States

**Keywords:** Aerobic threshold, Anaerobic threshold, Cardiorespiratory fitness, Oxygen consumption

## Abstract

**Background:**

The oxygen uptake efficiency slope (OUES) is considered a reliable indicator of cardiorespiratory fitness in young and clinical populations who cannot achieve maximal effort during a graded exercise test. However, OUES accuracy depends on the data points used for its calculation and it is still not clear if the submaximal OUES can accurately assess CRF in healthy young males.

**Objective:**

We investigated the association between peak oxygen uptake and peak and submaximal OUES, and the agreement between submaximal OUES and peak OUES in male adolescents and young adults.

**Methods:**

In this cross-sectional, observational study, fifty normal weight healthy participants (age 14–22 years, peak oxygen uptake 43.8 ± 7.3 mL·min^−1^·kg^−1^) performed a graded exercise test on a cycle ergometer and pulmonary gas exchange was assessed using breath-by-breath analysis. Peak oxygen uptake, and oxygen consumption at the aerobic and at the anaerobic threshold were determined as the 30-s average of the oxygen consumption values. Peak OUES (up to peak) and submaximal OUES (up to the aerobic and anaerobic thresholds) were calculated from the logarithmic relation between oxygen consumption and pulmonary ventilation.

**Results:**

Very strong correlations were observed between peak oxygen uptake and peak OUES (*r* = 0.80–0.88) while fair-to-very strong correlations were observed between the peak oxygen uptake and the two submaximal OUES (*r* = 0.32–0.81). The level of agreement between peak OUES and OUES up to the anaerobic threshold (*r* = 0.89–0.93; Typical percentage error 6%; Intraclass correlation coefficient = 0.89–0.93) was greater than the one between the peak oxygen uptake with OUES up to the aerobic threshold (*r* = 0.39–0.56; Typical percentage error 15%; Intraclass correlation coefficient = 0.38–0.56).

**Conclusions:**

. The peak OUES is a better indicator of aerobic fitness than the OUES up to the anaerobic threshold in healthy, young males. The OUES up to the anaerobic threshold is a valid alternative to peak OUES.

## Introduction

The oxygen uptake efficiency slope (OUES) is considered a reliable indicator of cardiorespiratory fitness (CRF) in young and clinical populations (Bongers et al., 2012). Derived from the linear relation between oxygen consumption (VO_2_) and the logarithm minute ventilation (VE) (VO_2_ = a × logVE + b), the OUES indicates how effectively oxygen is extracted from the air and transported to the working muscles during exercise (Baba et al., 1996; Yu et al., 2010). The OUES has gained popularity in the clinical field and in pediatric populations as it is considered a valid indicator of CRF in individuals that cannot achieve maximal oxygen uptake (VO_2max_) during a graded exercise test (Baba et al., 1999; Gavotto et al., 2020; Hossri et al., 2019; Yu et al., 2010). Moreover, compared to VO_2max_ the OUES is less subjected to human errors and its accuracy depends on the data points used for its calculation. Although the OUES calculated using all points up to peak VO_2_ (VO_2peak_) or VO_2max_ is the best indicator of CRF in children and adolescents, the submaximal OUES may be calculated using all points up to different percentages of exercise duration (Baba et al., 1996; Bongers et al., 2012; Bongers et al., 2011; Dias et al., 2017) or other individualized indices of fitness (Akkerman et al., 2010; Bongers et al., 2011; Breithaupt, Colley & Adamo, 2012; Drinkard et al., 2007; Marinov, Mandadzhieva & Kostianev, 2007; Sheridan et al., 2021). Research shows that OUES up to the aerobic threshold (AerT) is a useful and objective measure of CRF (Akkerman et al., 2010; Breithaupt, Colley & Adamo, 2012; Marinov, Mandadzhieva & Kostianev, 2007). However, the reliability of this index has been criticized as it should not be used as a replacement for VO_2max_ (Sheridan et al., 2021). Moreover, to our knowledge no research investigated the accuracy of the OUES in healthy young population when calculated up to the anaerobic thresholds (AnT) or to a respiratory exchange ratio (RER) of 1.0. The AnT is scarcely used in this population probably due to its association with metabolic acidosis and less with CRF (Meyer et al., 2005; Skinner & McLellan, 1980). However, the use of this biomarker could be of high relevance in the calculation of the OUES as it occurs immediately prior to the VO_2max,_ before muscle fatigue occurs.

This research aims at investigating the association between oxygen uptake and peak (up to VO_2peak_) and submaximal (up to the AerT and the AnT) OUES expressed in both absolute and relative terms in healthy, normal weight young males. Secondly, we aim at investigating the agreement between the peak OUES and different submaximal OUES (up to the AerT and the AnT) to identify which one should be used in this population. This research aims at providing an applicable framework for the use and the interpretation of the submaximal OUES in healthy, young males.

## Materials and Methods

### Study population

Fifty healthy, normal weight, and active adolescents and young adult males (14 to 22 years of age) were enrolled in this cross-sectional, observational multi-site study from North Carolina (USA) and Rome (Italy). The multi-site design caused body composition and aerobic fitness to be measured with different equipment (specified below). All study procedures were approved by the CAR-IRB - University of Rome “Foro Italico” Committee (Approval N° CAR 37/2020) and Appalachian State University Committee (IRB N° 17-0068 and 18-0147) performed in accordance with the Declaration of Helsinki 2013, with written informed consent obtained from all study participants/guardians. Normal weight individuals were recruited based on both body mass index (BMI) and percentage of fat mass (%FM) to include athletic individuals with high levels of BMI resulting from high fat free mass (FFM) and low %FM (Lopez-Sanchez et al., 2019; Provencher et al., 2018). The main inclusion criteria were a BMI < 85^th^ percentile specific to age and sex for adolescents and a BMI < 25 kg/m^2^ for adults. Athletic individuals with BMI ≥ 85^th^ percentile or BMI ≥ 25 kg/m^2^ but with %FM < 85^th^ percentile specific to age and sex were also included in the study (Borrud et al., 2010; McCarthy et al., 2006).

Exclusion criteria included having medical conditions including diabetes, heart, respiratory or renal disease and not taking any medications at the time of recruitment. Participants attended one visit lasting 90 min where anthropometric, body composition and cardiopulmonary exercise testing were performed between 8:00 to 11:00 AM or 4:00 to 6:00 PM. Participants were asked to avoid stimulants 4 h before the test, not perform moderate-to-vigorous exercise at least 12 h prior or eat large meals 4 h prior to the test.

### Anthropometrics and body composition

Body mass (BM) and height were measured using a scale and a stadiometer to the nearest 0.1 kg and 0.1 cm, respectively. BMI was calculated using the following formula *BMI = (kg/m*^*2*^*)* and body surface area (BSA) was calculated using the Haycock formula *BSA (m*^*2*^*) = 0.024265 × W*^*0.5378*^ *× H* ^*0.3964*^, where *W* represents body mass in kg and *H* is height in cm (Haycock, Schwartz & Wisotsky, 1978). Fat mass (FM) and FFM were assessed through air displacement plethysmography (BodPod technology; COSMED, Rome, Italy) using the Siri equation and predicted thoracic gas volume, a Dual-energy X-ray Absorptiometry (DEXA Horizon; Hologic, Marlborough, MA, USA), or a Bioelectrical Impedance Analysis (BIA AKERN 101 Anniversary; Pontassieve, FI, Italy). In all the analyses, participants were measured with minimal, tight fitting clothes and without shoes, sox and jewelry. BodPod measurements were performed with participants wearing a swimmer’s cap and seating in a relaxed position with hands flat on their thighs. DEXA measurements were performed with participants laying in a supine position with palms flat on the table and legs internally rotated. BIA measurements were taken with participants in a supine position with adhesive gel electrodes at defined sites on the dorsal surfaces of the hand, wrist, ankle, and foot.

### Cardiorespiratory fitness assessment

The cardiopulmonary exercise testing (CPET) was conducted on an electronically braked cycle ergometer (Lode Corrival; Lode BV, Groningen, Netherlands, or Monark 939 E; Monark Sport & Medical, Vansbro, Sweden) and by breath-by-breath method *via* a calibrated respiratory gas analysis system (K5 Wearable Metabolic Technology; Cosmed, Chicago, IL, USA, or Quark CPET; Cosmed, Rome, Italy) using a graded exercise test. The protocol included a 1-min resting period sitting on the cycle ergometer followed by a 1-min unloaded pedaling at 0 W, then the workload increased by 15, 20 or 25 W and participants were asked to keep a cadence of 60–70 revolutions per minute (rpm). The progression of the workload depended on the participant’s age and training status: 15 W per min for adolescents and young males (14–19 years) performing recreational sports, 20 W per min for adolescents and young males (14–19 years) performing competitive sports, 25 W per min for adult males (20–22 years) performing recreational sports.

The rate of perceived exertion was assessed using an OMNI scale 0–10 and was recorded at 15s prior to the end of every stage. The exercise was considered valid when one of the following criteria were met: the participant achieved volitional exhaustion or a cadence of 50 rpm, a value of 10 on the OMNI scale 0–10, the 90% of the predicted maximal heart rate (beats/min) or a respiratory exchange ratio (RER) equal to 1.1.

### Gas exchange thresholds

VO_2_ (mL·min^−1^·kg^−1^) at the AerT (VO_2AerT_) was graphically determined using the ventilatory equivalent for O_2_ (VE/VO_2_) method as primary criterion and the V-slope method as a secondary criterion. VO_2_ (mL·min^−1^·kg^−1^) at the AnT (VO_2AnT_) was graphically determined using the ventilatory equivalent for carbone dioxide (VE/VCO_2_) method as primary criteria and VE *vs* VCO_2_ method as a secondary criterion (Meyer et al., 2005). The VO_2AerT_ and the VO_2AnT_ were calculated as the 30-s average of the VO_2_ values at the point of the threshold. Peak Oxygen Uptake (VO_2peak_) was the 30-s average of the highest VO_2_ during the last minute of the exercise test before the test was terminated.

### Oxygen uptake efficiency slope (OUES)

OUES was determined by the linear regression when each participant’s VO_2_ (mL·min^−1^) was plotted against the logarithm of their V_E_ (L·min^−1^). In accordance with the original equation VO_2_ = a log VE + b, the ‘*a*’ coefficient was defined as the oxygen uptake efficiency slope (Baba et al., 1996). In the OUES calculation, all data points up to AerT, up to AnT (submaximal OUES), and up to peak of exercise (peak OUES) were included in the analyses. To avoid possible irregular breathing patterns, data from the 2-min resting period, from the first minute of exercise, and from a plateau in oxygen consumption were excluded from the calculation of the OUES (Akkerman et al., 2010; Wasserman et al., 1999). The OUES values expressed in absolute terms and relative to body mass, BSA and FFM were used for further analysis.

### Quality control

The principal investigators at the two sites completed a 3-month training period prior to the start of the project to ensure consistency with data collection and data reduction. BodPod and BIA body composition measurements were taken twice, and results were averaged, DEXA measurement was performed once to reduce exposure to radiation. Body composition was assessed using the technology available in the respective laboratories. Prior to each CPET, a turbine calibration (using a 3-L syringe), a two-point gas calibration (16.00% and 20.93% O2; 5.0% and 0.04% CO2), a CO2 scrubber calibration (0.00% CO2), and a delay calibration were performed on both the Quark and K5 equipment according to the manufacturer’s recommendation (Guidetti et al., 2018). Heart rate during the exercise test was recorded using GARMIN HR chest belt (GARMIN, Olathe, KS, USA). Raw breath-by-breath data from the CRF assessment were reduced using a six-breaths moving average (smoothing) and averaged at 10 s on the OMNIA (COSMED, Rome, Italy) software and imported on a shared separate Excel file for further analysis. The AerT, AnT and VO_2peak_ were determined separately by the two principal investigators together with an experienced exercise physiologist, and in case it varied more than 30 s, the opinion of a third exercise physiologist was considered.

### Statistical analysis

Differences between OUES values (up to AerT, up to AnT and peak) were identified using repeated measures analysis of variance (RM ANOVA) and if significance was found, a Bonferroni *post hoc* test was conducted to determine where the significance lied between the group comparisons. A Pearson’s correlation coefficient (*r*) was calculated to examine the association between the VO_2AerT_, VO_2AnT_ and VO_2peak_ and peak OUES (up to VO_2peak_) and submaximal OUES (up to AerT and up to AnT) in absolute and relative terms. The intervals used to interpret the Pearson’s correlation coefficient r were: ≥0.8 “very strong”, 0.6–0.8 “moderately strong”, 0.3–0.5 “fair”, <0.3 “poor” (Chan, 2003). Agreements between peak OUES and the two submaximal OUES were assessed by ordinary least products (OLP) regression analysis. In this analysis the coefficients of determination (R^2^) and slope and intercept with the 95% of confidence intervals (95% CI) were calculated to verify fixed and proportional biases. The hypothesis of proportional and fixed bias was rejected when the 95% CI contained the value 1 for the slope and the 0 for the intercept. The differences between peak OUES and the two submaximal OUES were reported as mean and range values. The validity of these methods was also assessed by comparing peak OUES *vs* the two submaximal OUES with a paired samples t-test. “Typical percentage error” (TE) was used to calculate the error of the two submaximal OUES. An additional parameter for criterion validity was the intraclass correlation coefficient (ICC) which allowed to compare peak OUES with the two submaximal OUES. The intervals used to interpret the ICC were: 0.75–1 “excellent”, 0.60–0.75 “good”, 0.40–0.60 “fair”, <0.40 “poor” (Cicchetti & Sparrow, 1981; Perinetti, 2018). Bland-Altman plots were used to determine the 95% CI between peak OUES and the two submaximal OUES (Bland & Altman, 1986). All analyses were performed using SPSS software, (SPSS Inc., IBM, Chicago, IL, USA). The level of significance was set at *p* < 0.05. Results are expressed as mean ± standard deviation.

## Results

Participants’ descriptive characteristics are shown in Table 1. Gas exchange and performance parameters at the AerT and AnT are reported in Table 2. The AerT and AnT occurred at the 53 ± 9% and 78 ± 9% of VO_2peak_, respectively. Differences between the three OUES calculations are described in Table 3. No significant differences were observed between OUES up to AerT, OUES up to AnT and peak OUES, expressed in both absolute terms and relative to BM, FFM and BSA. No significant difference between the 15, 20 and 25 W/min protocols was observed in time to exhaustion (12.3 ± 2.4 min; 12.4 ± 2.3 min; 10.6 ± 1.4 min; *p* = 0.106, respectively) and RER (1.15 ± 0.11; 1.13 ± 0.05; 1.21 ± 0.06; *p* = 0.160, respectively) at peak exercise indicating appropriate selection of the exercise protocol.

**Table 1:** Participants’ descriptive characteristics.

	Mean ± SD
Age (years)	16.9 ± 2.6
Height (cm)	176.3 ± 7.0
Body mass (kg)	68.0 ± 11.4
BMI (kg/m^2^)	21.7 ± 2.9
BSA (m^2^)	1.82 ± 0.18
FFM (kg)	56.3 ± 8.5
FM (kg)	11.7 ± 4.6
%FFM (%)	83.1 ± 5.2
%FM (%)	16.9 ± 5.2

**Note:**

Data are presented as mean ± standard deviation. BMI, body mass index; BSA, body surface area; FFM, fat free mass; FM, fat mass, %FFM, percentage FFM, %FM, percentage FM.

**Table 2:** Physiological parameters obtained from the CPET at the aerobic (AerT) and anaerobic (AnT) threshold, and at peak oxygen consumption (peak).

	AerT Mean ± SD	AnT Mean ± SD	Peak Mean ± SD
HR (beat·min^−1^)	128.5 ± 17.0	161.1 ± 14.4	186.3 ± 10.1
RER	0.88 ± 0.06	1.02 ± 0.06	1.16 ± 0.10
Power (W)	82 ± 31	147 ± 40	208 ± 49
VO_2_ (mL·min^−1^)	1,569 ± 468	2,297 ± 574	2,961 ± 646
VO_2_/BM (mL·min^−1^·kg^−1^)	23.4 ± 6.5	34.1 ± 7.5	43.8 ± 7.3
VO_2_/FFM (mL·min^−1^·kg^−1^)	28.2 ± 8.1	41.2 ± 9.4	52.7 ± 8.8
VO_2_/BSA (mL·min^−1^·m^−2^)	863 ± 230	1,262 ± 269	1,624 ± 273
VE (L·min^−1^)	37.9 ± 12.1	63.4 ± 16.7	117.2 ± 30.7

**Note:**

Data are presented as mean ± standard deviation. HR, heart rate; RER, respiratory exchange ratio; VE, ventilation; VO_2_, oxygen consumption in absolute terms; VO_2_/BM, oxygen consumption relative to body mass (kg); VO_2_/BSA, oxygen consumption relative to body surface area (m^2^); VO_2_ /FFM, oxygen consumption relative to fat free mass (kg).

**Table 3:** Values of the oxygen uptake efficiency slope calculated up to the aerobic (AerT) and anaerobic (AnT) threshold, and up to peak oxygen consumption (peak).

	Up to AerT Mean ± SD	Up to AnT Mean ± SD	Peak Mean ± SD	F	Partial eta squared
OUES	2,907 ± 656	3,008 ± 575	3,022 ± 605	1.96	0.04
OUES/BM	43.3 ± 9.3	44.8 ± 7.5	45.0 ± 8.0	1.90	0.04
OUES/FFM	52.0 ± 10.6	53.9 ± 9.1	54.2 ± 9.8	2.17	0.04
OUES/BSA	1,599 ± 316	1,655 ± 255	1,663 ± 275	1.99	0.04

**Note:**

Data are presented as mean ± standard deviation. OUES, OUES in absolute terms; OUES/BM, OUES relative to body mass (kg); OUES/BSA, OUES relative to body surface area; OUES/FFM, OUES relative to fat free mass (kg).

Oxygen consumption showed higher correlations with peak OUES (*r* = 0.79–0.89) compared to the two submaximal OUES (*r* = 0.32–0.87) (Table 4). The highest correlations were observed between peak OUES and VO_2AnT_ in both absolute and relative terms (*r* = 0.85–0.89).

**Table 4:** Correlations between VO2 at the AerT, AnT and peak with the OUES up to AerT, AnT and peak.

	OUES up to AerT	OUES up to AnT	Peak OUES
VO_2AerT_	0.51	0.74	0.82
VO_2AnT_	0.55	0.87	0.89
VO_2peak_	0.57	0.81	0.88
	OUES up to AerT/BM	OUES up to AnT/BM	Peak OUES/BM
VO_2AerT_/BM	0.42	0.71	0.81
VO_2AnT_/BM	0.40	0.80	0.85
VO_2peak_/BM	0.33	0.66	0.81
	OUES up to AerT/BSA	OUES up to AnT/BSA	Peak OUES/BSA
VO_2AerT_/BSA	0.37	0.68	0.79
VO_2AnT_/BSA	0.37	0.80	0.85
VO_2peak_/BSA	0.32	0.66	0.80
	OUES up to AerT/FFM	OUES up to AnT/FFM	Peak OUES/FFM
VO_2AerT_/FFM	0.46	0.74	0.83
VO_2AnT_/FFM	0.43	0.83	0.87
VO_2peak_/FFM	0.33	0.69	0.82

**Note:**

/BM, relative to body mass; /BSA, relative to body surface area; /FFM, relative to fat free mass; *V*O_2AerT_, oxygen consumption at the AerT; VO_2AnT,_ oxygen consumption at the AnT; VO_2peak_, oxygen consumption at peak exercise.

**Figure 1:**
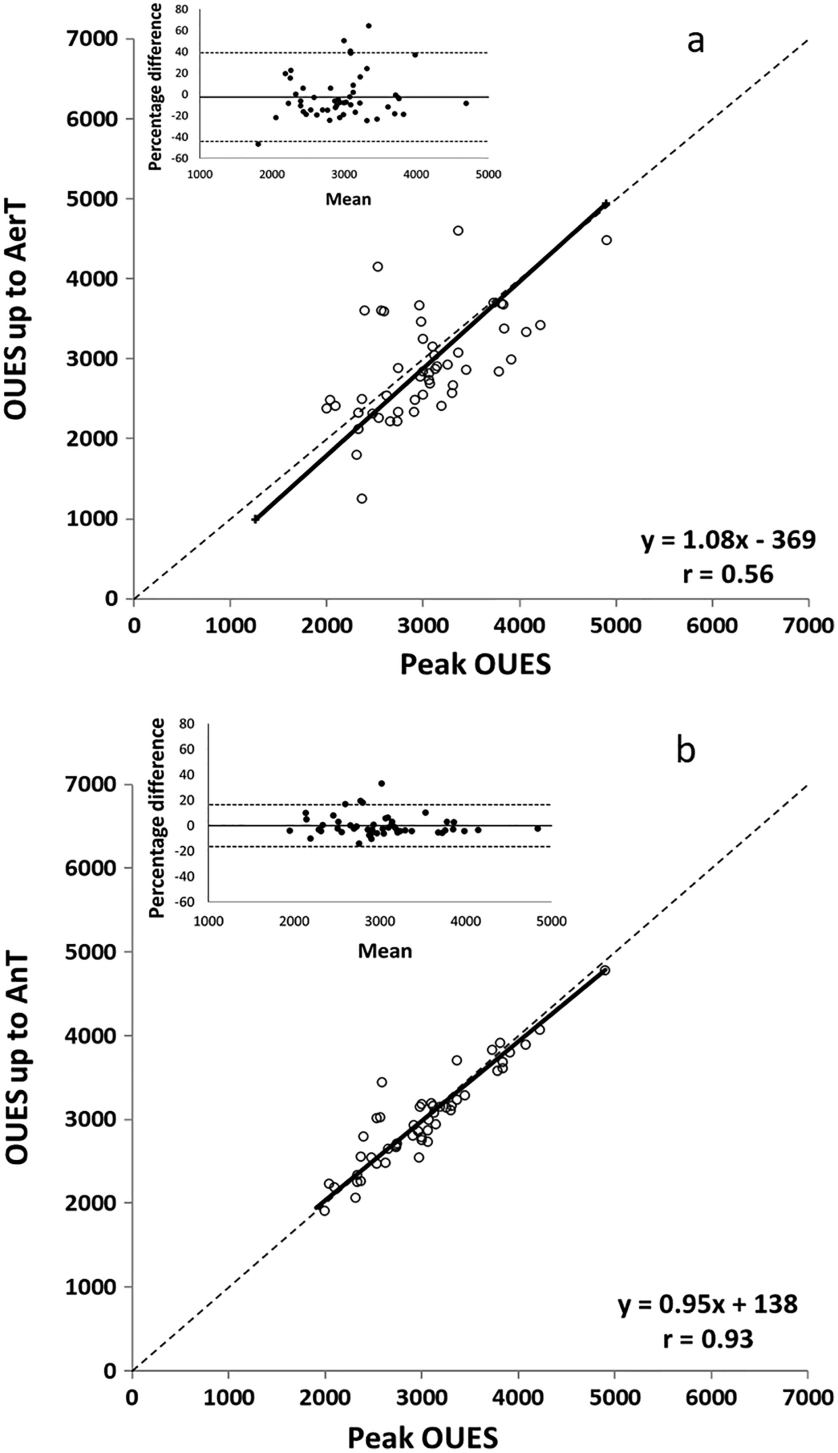
OLP regression and difference (Bland Altman) plots of peak OUES and OUES up to AerT (A) and of peak OUES and OUES up to AnT (B). The OLP regression plots are described by the linear regression (solid line), the identity (dashed line), the equation and the correlation coefficient (*r*). Bland Altman plots with the percentage mean difference (solid lines) and the 95% CI (dashed lines) are shown in the upper-left panel.

The level of agreement between the peak OUES and the two submaximal OUES is reported in Table 5. Peak OUES showed fair-to-moderate correlations with OUES up to AerT (*r* = 0.39–0.56) and very strong correlations with OUES up to AnT (*r* = 0.89–0.93) when expressed in both absolute and relative terms (Chan, 2003). Slope and intercept values always included the 1 and the 0, respectively. Mean percentage differences were −3.8% for peak OUES and OUES up to AerT, and −0.5% for peak OUES and OUES up to AnT. No significant differences were observed between peak OUES and the two submaximal OUES. The TE for the OUES up to AerT (15%) was higher than the acceptable 10% limit while the TE for the OUES up to AnT (6%) was considered acceptable (Alexander, Tropsha & Winkler, 2015). Intraclass correlation coefficient values were poor-to-fair for OUES up to AerT (ICC = 0.38–0.56) and excellent for OUES up to AnT (ICC = 0.89–0.93) (Cicchetti & Sparrow, 1981; Perinetti, 2018). The OLP regression analysis and Bland-Altman plots between peak OUES and the two submaximal OUES are graphically shown in Fig. 1. The Bland-Altman plot reporting the peak OUES and the OUES up to AerT shows a mean percentage difference of −2.40% and the 95% CI of −43.97% and 39.18%. The Bland-Altman plot reporting the peak OUES and the OUES up to AnT shows a mean percentage difference of 0.00% and the 95% CI of −16.37% and 16.37%.

**Table 5:** Agreement between peak OUES and the two submaximal OUES calculation methods (up to AerT and AnT).

	R^2^	Slope (95% CI)	Intercept (95% CI)	Mean diff. (min to max)	*p*	TE (%)	ICC (95% CI)
Peak OUES - OUES up to AerT	0.32	1.08 [0.86 to 1.37]	−369 [−1,247 to 323]	−115 [−1,105 to 1,625]	0.18	15	0.56 [0.34 to 0.72]
Peak OUES/BM - OUES up to AerT/BM	0.21	1.16 [0.90 to 1.49]	−8.9 [−24.0 to 2.9]	−1.7 [−18.8 to 25.7]	0.19	15	0.46 [0.21 to 0.65]
Peak OUES/FFM - OUES up to AerT/FFM	0.21	1.08 [0.84 to 1.39]	−6.5 [−23.5 to 6.7]	−2.2 [−21.3 to 28.8]	0.16	15	0.45 [0.20 to 0.65]
Peak OUES/BSA - OUES up to AerT/BSA	0.15	1.15 [0.88 to 1.50]	−310 [−887 to 133]	−64 [−658 to 828]	0.17	15	0.38 [0.12 to 0.60]
Peak OUES - OUES up to AnT	0.86	0.95 [0.85 to 1.06]	138 [−190 to 433]	−14 [−416 to 858]	0.66	6	0.93 [0.87 to 0.96]
Peak OUES/BM - OUES up to AnT/BM	0.81	0.94 [0.83 to 1.06]	2.7 [−5.4 to 7.6]	−0.2 [−5.6 to 12.1]	0.66	6	0.90 [0.83 to 0.94]
Peak OUES/FFM - OUES up to AnT/FFM	0.83	0.93 [0.82 to 1.05]	3.7 [−2.8 to 9.4]	−0.3 [−6.2 to 14.3]	0.65	6	0.91 [0.84 to 0.95]
Peak OUES/BSA - OUES up to AnT/BSA	0.79	0.93 [0.81 to 1.06]	113 [−106 to 304]	−8 [−207 to 466]	0.66	6	0.89 [0.81 to 0.93]

**Note:**

OUES is expressed in both absolute and relative terms (/BM, body mass; /FFM, fat free mass; /BSA, body surface area). ICC, intraclass correlation coefficient; R^2^, coefficient of determination; TE, typical percentage error.

## Discussion

This study aims at investigating the association between oxygen uptake and OUES calculated up to the AerT, AnT and peak exercise in healthy, normal weight young males. Secondly, we aim at investigating the agreement between the peak OUES and the two submaximal OUES to identify which one should be used in this population. The present study indicates that the peak OUES is a better indicator of aerobic fitness then the OUES up to AnT and the OUES up to AerT in healthy, normal weight male adolescents and young adults. Moreover, the OUES including all data points up to AnT shows higher levels of agreement with peak OUES compared to the OUES up to AerT.

Observing the correlation between oxygen consumption and peak and submaximal OUES, our results suggest that the peak OUES can be considered the best indicator of aerobic fitness in this population while the OUES up to AerT is the least accurate indicator (Table 4). Peak OUES showed moderate-to-very strong correlations with VO_2peak_, VO_2AerT_ and VO_2AnT_ (*r* = 0.79–0.89) while the OUES up to AerT showed only fair correlations (*r* = 0.32–0.57) (Chan, 2003). Differently to what observed in our research, previous studies in healthy and obese children reported moderate-to-strong correlations between the OUES up to AerT and the VO_2peak_ (*r* = 0.71–0.88) (Akkerman et al., 2010; Breithaupt, Colley & Adamo, 2012). Moreover, we observed fair correlations between the OUES up to AerT and the VO_2AerT_ while previous studies reported high correlations between the two indicators of aerobic fitness (Akkerman et al., 2010). These differences may be explained by the fact that the participants recruited in previous research had different age and level of fitness compared to the one in our study. It is well known that OUES depends on both mathematical and physiological factors. From the mathematical standpoint, the most accurate OUES values are obtained when all datapoints measured during the CPET are used to calculate the OUES (Baba et al., 1996), which explains why the peak OUES is a better indicator of aerobic fitness than the OUES up to AerT in our results. From the physiological standpoint, these difference can be explained by the fact that pediatric and clinical populations achieve the AerT at a higher percentage of VO_2peak_ (Meyer et al., 2005) which leads to the inclusion of a larger amount of data points in the calculation of the OUES. This causes the OUES up to AerT to be better correlated to VO_2AerT_ and VO_2peak_ in pediatric and clinical populations compared to healthy and adult individuals who achieve the AerT at a lower percentage of VO_2peak_ (Akkerman et al., 2010; Breithaupt, Colley & Adamo, 2012). These results highlight the importance of considering age and fitness level in the interpretation of the OUES as both AerT and AnT may occur at higher percentages of the VO_2peak_ in children compared to adults, and in individuals with low and normal levels of aerobic fitness compared to fit individuals (Meyer et al., 2005).

To the best of our knowledge, previous studies only considered the AerT and the lactate inflection point as individualized exercise intensities to calculate the OUES (Akkerman et al., 2010; Bongers et al., 2011; Breithaupt, Colley & Adamo, 2012; Drinkard et al., 2007; Marinov, Mandadzhieva & Kostianev, 2007; Sheridan et al., 2021). Therefore, the strength of the current study is the use of the AnT as an individualized and submaximal parameter for the calculation of the OUES. To investigate the validity of different submaximal OUES indices in our sample, the OUES up to AerT and up to AnT were compared with the peak OUES. Our results showed that the OUES up to AnT is a better indicator of aerobic fitness than the OUES up to AerT as shown by the higher coefficient of correlation (very strong *vs* fair-to-moderate), an acceptable TE (6% *vs* 15%) (Alexander, Tropsha & Winkler, 2015), and higher levels of agreement (mean percentage difference −0.5% *vs* −3.8%, and excellent *vs* poor-to-fair ICC) (Cicchetti & Sparrow, 1981; Perinetti, 2018). This finding has important implications as, although no significant differences were observed between peak OUES and submaximal OUES parameters (Table 3), the OUES up to AerT may not be considered an accurate indicator of aerobic fitness in healthy, normal weight male adolescents and young adults. Our results are in accordance with a recent investigation which observed that the OUES up to AerT does not replace VO_2max_ in the assessment of CRF in male adolescents (Sheridan et al., 2021). However, it is important to consider that the OUES up to AerT can be a useful and objective measure of cardiorespiratory function when testing children and clinical populations (Akkerman et al., 2010; Breithaupt, Colley & Adamo, 2012; Marinov, Mandadzhieva & Kostianev, 2007). In addition to the mathematical and physiological reasons previously discussed, methodological reasons may help explain why the OUES up to AnT should be preferred to the OUES up to AerT in the assessment of the aerobic fitness in individuals that are able to achieve the AnT. Previous research questioned the accuracy, the intra- and inter-observer reliability, and the repeatability of the AerT (Meyer et al., 2005; Zignoli et al., 2021), while the AnT is of easier determination due to the marked hyperventilation at the onset of metabolic acidosis, and to its correspondence to a RER of 1.00 (Meyer et al., 2005; Solberg et al., 2005; Zignoli et al., 2021). These results are relevant when considering the safety of the participant during a CPET. In fact, accurate OUES values can be obtained in individuals that are able to achieve a RER of 1.00 ad that cannot or should not exercise to vigorous exercise intensities.

Lastly, similar correlations were observed between the peak OUES and VO_2_ at the AerT, AnT, and peak when expressed relative to body mass (*r* = 0.81–0.85), FFM (*r* = 0.82–0.87) and BSA (*r* = 0.79–0.85). This result can be explained by the fact that normal weight individuals of the same sex also have similar body mass, FFM and BSA. Therefore, small changes in body composition only minimally affects OUES values. The use of body composition and anthropometric measures to study differences in the OUES should be considered only when more heterogeneous population are investigated (Breithaupt, Colley & Adamo, 2012). Future research should investigate whether the methodological and conceptual approach used in this study could provide valuable information in the analysis of the OUES in healthy adolescent and adult females.

A limitation of this study is the use of different body composition measurements. However, BOD POD and bioelectrical impedance analysis provide comparable results with DEXA in young man (Burns, Fu & Constantino, 2019). Another limitation is the relatively small sample size composed of adolescents and young adults. However, only normal weight, healthy males were recruited in this study. Age, sex, and health conditions can affect OUES (Akkerman et al., 2010; Breithaupt, Colley & Adamo, 2012; Drinkard et al., 2007; Hossri et al., 2019; Marinov, Mandadzhieva & Kostianev, 2007) and the ability to achieve maximal effort (Poole & Jones, 2017). Therefore, all participants recruited for this study were male, healthy, and classified as normal weight. Moreover, all participants were able to achieve both the AerT and the AnT. Additionally, to control for the effect of age on the OUES, preliminary analyses showed that the peak OUES relative to body mass, BSA and FFM, peak RER, and VO_2peak_ relative to body mass were not different between adolescents and young adults (*p* = 0.51, 0.59, 0.89, 0.45, 0.79 respectively). These results indicate that participant in both groups achieved similar peak metabolic effort and similar fitness level. Therefore, for all further analyses, we combined our participants into respective groups. However, future studies should examine whether the method used in this study will provide similar results in healthy female adolescents and young adults and obese individuals.

## Conclusions

The OUES up to AnT could be a valid alternative to the peak OUES in healthy, normal weight male adolescents and young adults. This finding has important safety implications as we can accurately assess cardiorespiratory fitness interrupting a graded exercise test at the onset of metabolic acidosis (RER of 1.00) in clinical populations that can exercise at moderate-to-vigorous exercise intensities but that cannot or should not exert at severe or maximal intensities. Therefore, this study provides an applicable framework for the use and the interpretation of the OUES in this population.

## Supplemental Information

10.7717/peerj.13709/supp-1 Supplemental Information 1 Anonymised raw data for CPET Parameters, OUES, and VO2 (Tables 2–5 and Figure 1).

10.7717/peerj.13709/supp-2 Supplemental Information 2 OLP regression (OUES peak - OUES AerT). The OLP regression plots are described by the linear regression (solid line), the identity (dashed line), the equation and the correlation coefficient (r).

10.7717/peerj.13709/supp-3 Supplemental Information 3 OLP regression (OUES peak - OUES AnT). The OLP regression plots are described by the linear regression (solid line), the identity (dashed line), the equation and the correlation coefficient (r).

10.7717/peerj.13709/supp-4 Supplemental Information 4 BlandAltman (OUESpeak - OUES AerT). Bland Altman plots with the percentage mean difference (solid lines) and the 95% CI (dashed lines) are shown in the upper-left panel.

10.7717/peerj.13709/supp-5 Supplemental Information 5 BlandAltman (OUESpeak - OUES AnT). Bland Altman plots with the percentage mean difference (solid lines) and the 95% CI (dashed lines) are shown in the upper-left panel.

10.7717/peerj.13709/supp-6 Supplemental Information 6 Raw data: Characterization of the study sample with CPET information.

## Additional Information and Declarations

### Data Availability

The following information was supplied regarding data availability: The OLP regression analyses, Bland - Altman analyses, characterization of the study sample, anonymised raw data for CPET Parameters, OUES, and VO_2_ (Tables 2–5 and Figure 1) are available in the Supplemental Files.
